# Cognitive predictors of treatment outcome for exposure therapy: do changes in self-efficacy, self-focused attention, and estimated social costs predict symptom improvement in social anxiety disorder?

**DOI:** 10.1186/s12888-019-2054-2

**Published:** 2019-02-22

**Authors:** Isabel L. Kampmann, Paul M. G. Emmelkamp, Nexhmedin Morina

**Affiliations:** 1 Department of Clinical Psychology, University of Amsterdam, Nieuwe Achtergracht 129 B, 1018 WS Amsterdam, The Netherlands; 2 Department of Psychology, Westfälische Wilhelms-University Münster, Fliednerstraße 21, 48149 Münster, Germany; 3 HSK Groep Woerden, Polanerbaan 3, 3447 GN Woerden, The Netherlands

**Keywords:** Virtual reality, Exposure therapy, Cognitive predictors

## Abstract

**Background:**

Cognitions play an important role in the development and maintenance of social anxiety disorder (SAD).

**Methods:**

To investigate whether changes in cognitions during the first six sessions of exposure therapy are associated with treatment outcome, we assessed reported self-focused attention, self-efficacy in social situations, and estimated social costs in 60 participants (*M*_age_ = 36.9 years) diagnosed with SAD who received in vivo or virtual reality exposure therapy.

**Results:**

Patients demonstrating a greater decrease in estimated social costs during treatment reported greater improvement of their social anxiety symptoms following both forms of exposure therapy. While changes in self-focused attention and social self-efficacy during treatment were significantly associated with treatment outcome when examined individually, these changes did not significantly predict symptom improvement beyond social costs.

**Conclusions:**

Changes in estimated social costs during treatment are associated with improvement of social anxiety symptoms after exposure therapy. Future research needs to further investigate estimated social costs as a predictor in relation to other cognitive variables.

**Trial registration:**

NCT01746667; www.clinicaltrials.gov, November 2012, retrospectively registered.

## Background

Social anxiety disorder (SAD) refers to the fear of one or more social situations in which one can behave embarrassingly or be scrutinized by others [1]. Cognitive models of SAD assume that an individual’s negative beliefs related to social situations as well as the fear of behaving inappropriately and the subsequent consequences play an important role in the development and maintenance of the disorder [2]. Although exposure therapy, a psychological intervention that focusses on fear-related overt behaviour, does not directly address maladaptive cognitions, research suggests that this effective treatment produces similar changes in cognitions as cognitive therapy or cognitive behavioural therapy (see [3–5]). This suggests that although exposure therapy does not explicitly target cognitions during treatment, changes in cognitions can occur. However, it is unclear whether changes in SAD-related cognitions are associated with treatment outcome of pure exposure therapy. In this article, we examine the role of three cognitive variables in predicting treatment outcome in exposure therapy: self-focused attention, self-efficacy in social situations, and estimated social costs.

Self-focused attention in social situations, where an individual shows increased vigilance for internal stimuli (e.g., cognitions, emotions, physiological reactions), has been suggested to play an important role in the maintenance of SAD [2, 6]. Research indicates that self-focused attention is associated with higher social anxiety levels and biased self- and performance judgements in SAD [7]. Moreover, in cognitive behavioural therapy, higher levels of maladaptive attentional focus predicted slower symptom improvement [8] and a decrease in self-focused attention predicted and mediated reductions in social anxiety (see [9], for a review). Hofmann [10] showed that exposure therapy, although it does not explicitly target cognitions, can lead to reduced self-focused attention in patients with SAD after treatment. However, it remains unclear whether changes in self-focused attention are associated with treatment outcome in pure exposure therapy.

Additionally, social anxiety has been found to be associated with low levels of self-efficacy in social situations, which refers to one’s belief in his or her own ability to achieve certain goals while interacting with other individuals. A lack of self-efficacy might facilitate the use of dysfunctional coping strategies when experiencing anxiety in social situations [11]. Research on cognitive behavioural therapy indicates that in-treatment changes in self-efficacy in social situations [12, 13] and in therapy context [14] are associated with treatment outcome. Yet, there is a lack of research as to whether changes in self-efficacy during exposure treatment that does not explicitly targets cognitions, are associated with treatment outcome.

The expected negative consequences of social behaviour, namely the social costs, might also play an important role in maintaining SAD, as suggested by Foa and Kozak [15]. More specifically, individuals with SAD tend to overestimate the costs of negative social incidents [16]. Changes in estimated social costs during treatment have been positively associated with treatment outcome in cognitive therapy [16], cognitive behavioural therapy [17–19], drug treatment [16], and exposure therapy [20]. However, current literature is inconclusive regarding the role of estimated social costs as a predictor of treatment outcome in SAD. While some studies found that decreased estimated social cost mediated improvement during cognitive behavioural therapy [18, 19, 21] and exposure therapy [19], others found contradicting results. More specifically, Calamaras and colleagues [17] concluded from their study, that although change in cost bias mediated treatment outcome of cognitive behavioural therapy, cost bias at midtreatment did not significantly predict treatment outcome. Additionally, decreases in estimated costs did not precede decreases in anxiety levels during exposure therapy [20]. The association between estimated social costs and social anxiety levels might also depend on a temporal component, as suggested by Gregory and colleagues [22] who found cost bias to predict social anxiety levels in early but not in final stages of treatment. Yet, the results from studies on social cost bias in exposure therapy were limited to exposure therapy administered in a group format [19] and public speaking anxiety only, and hence neglected other social situations that are relevant for SAD patients with heterogeneous fears [20]. However, research suggests that working mechanisms can vary across treatment formats. A study by Hedman et al. [23] indicates that although symptom improvement during cognitive therapy was mediated by decreases in self-focused attention in both group and individually administered treatment, decreases in avoidance mediated effects of individual cognitive therapy only, and decreases in anticipatory and post-event processing mediated effects of group therapy only. Therefore, research that addresses various social fears is needed to investigate the role of social cost bias in exposure treatment for SAD and to investigate whether changes in estimated social costs during individual exposure therapy are associated with treatment outcome.

The aim of the current study was to examine the association between change in SAD-related cognitions and treatment outcome in individually administered exposure therapy (without any cognitive components) for SAD. The association between change in self-focused attention, self-efficacy in social situations, and estimated social costs and change in social anxiety symptoms was evaluated across both in vivo exposure therapy (iVET) and virtual reality exposure therapy (VRET) for SAD. We expected that decreases in self-focused attention and estimated social costs, and an increase in self-efficacy during treatment would be associated with treatment outcome across both exposure treatments. If changes in these cognitions are associated with treatment outcome, these findings might form the base for further research on working mechanisms of exposure therapy. Eventually, this research can help to improve the efficacy of exposure treatments by informing us on which cognitions we need to focus on during therapy and which related changes we need to facilitate.

## Methods

### Participants

Sixty participants (*M*_age_ = 36.9 years, age range: 18–65 years, 63.3% women) meeting the diagnostic criteria for a primary diagnosis of SAD [24] were included in the present study after being recruited via online and newspaper advertisements, and project related websites. Of these participants, 90% reported Dutch as native language, 48.3% high, 46.7% middle, and 5% low education, 60% had a paid employment status, and 50% were married. Furthermore, 11.7% fulfilled the criteria for a comorbid anxiety disorder, 10% for a depressive disorder, and 26.7% for a avoidant personality disorder. For more details on sample characteristics and a participant flow-chart, see Kampmann et al. [25].

### Measures

#### Screening

As a first step in the screening procedure, social anxiety levels were assessed using an online version of the Social Interaction Anxiety Scale (SIAS; [26]), a 20-item self-report measure rated on a 5-point Likert scale. If individuals had a mean SIAS score of ≥29, they were invited to an in-person interview in which a psychologist administered the Structured Clinical Interview for DSM-IV-TR Axis I Disorders (SCID-I; [27]) to assess the diagnoses of SAD and other mental disorders.

#### Outcome measure

The Liebowitz Social Anxiety Scale-Self Report (LSAS-SR; [28]) was used to assess social anxiety symptoms at pre- and postassessment. The LSAS-SR contains 24 items on fear and avoidance in social situations that are rated on a 4-point Likert scale. The LSAS-SR has good psychometric properties [29] and was assessed at pre- and postassessment. The internal consistency in the present study was high (Cronbach’s α = .90–.96).

#### Potential predictors of SAD symptom improvement

The extent to which participants focused on themselves during in vivo or virtual reality exposure exercises was assessed with the self-focus subscale of the Focus of Attention Questionnaire [30]. The internal consistency has been reported to be good (Cronbach’s α = .76; [31]) and was good in the present study (Cronbach’s α = .76–.81). The FAQ is a 10-item self-report measure rated on a 5-point Likert scale with higher scores on the self-focus subscale indicating greater focus of attention on the self. In line with the goal of the FAQ to assess focus of attention during exposure exercises, this questionnaire was administered after Session 3 (Time 1), which was the first exposure session, and after Session 6 (Time 2).

Self-efficacy was assessed with the Self-Efficacy for Social Situations Scale (SESS; [13]), a 9-item self-report measure rated on a 10-point Likert Scale with higher scores indicating higher self-efficacy for social situations. The SESS assesses beliefs on self-efficacy for social skills, cognitive coping, and affective coping. The SESS was assessed before Session 1 (Time 1) and before Session 7 (Time 2). The internal consistency has been reported in the literature to be high (Cronbach’s α = .82; [13]) with Cronbach’s α = .75–.81 in the current study.

The estimated costs related to social situations were assessed using the social event subscale of the Social Costs Questionnaire (SCQ; [18]) which is a 20-item self-report questionnaire (10 items on social performance situations and 10 items on social nonperformance situations) rated on a 9-point Likert scale ranging from 0 (not at all bad) to 8 (extremely bad). In line with Foa et al. [18], a mean score of performance and nonperformance items was calculated after detecting high correlations between both subscales at all assessment points (*r* = .75–.86). The internal consistency across both subscales was excellent (Cronbach’s α = .81–.93). The SCQ was assessed at preassessment (Time 1) and before Session 7 (Time 2) because treatment completers were those participants who completed as least six treatment sessions. All completers therefore received Session 1 (therapy rationale), Session 2 (exposure hierarchy), and then four exposure sessions (Session 3–6, in case they continued treatment) or three exposure sessions (Session 3–5) and a closing session containing relapse prevention and evaluation of the treatment (content of Session 10) in case they were going to stop treatment after six sessions. Therefore, completers could only differ in a maximum of one exposure session. See 2.3. and 2.4. for more information on treatment sessions and assessments.

### Treatment

Both treatments, VRET and iVET, were based on previous treatment protocols for SAD [32, 33]. However, to investigate the effects of pure exposure on cognitions, we only applied behavioural elements and abandoned all cognitive elements of the treatment protocol. Treatment in both conditions consisted of ten semi-weekly sessions with a duration of 90 min (including 60 min exposure) and was provided by therapists (clinical psychologists and last-semester master’s students) who had undergone training in administering both treatments. In Session 1 and 2, the therapy rationale was discussed and a hierarchy of social situations (available virtual situations for VRET and participant’s individual social situations for iVET) was obtained. Session 3 through 9 contained virtual reality or in vivo exposure, respectively. Session 10 comprised relapse prevention and evaluation of the treatment.

#### VRET

Participants receiving VRET were exposed to computer-simulated social situations by means of a head-mounted display in a laboratory at the University of Amsterdam. During VRET, participants were instructed to engage in verbal interaction with virtual humans in one-to-one or group situations to confront their social fears. These virtual scenarios covered the following social situations: public speaking, small-talk with a stranger, buying and returning clothes, attending a job interview, being interviewed by journalists, having dinner in a restaurant with a friend, and having a blind date [34]. Verbal interaction was implemented using semi-structured dialogues that were controlled by the therapist [34] who could also tailor exposure exercises to the individual participant by manipulating certain scenario characteristics: dialogue style (friendly or unfriendly), gender of virtual human, number of virtual humans present in the virtual world, dialogue topic’s degree of personal relevance, and virtual human’s gestures (i.e., gaze direction and posture). A nVisor SX60 head-mounted display with 1280 × 1024 pixel resolution, a stereographic projection, and a 60° diagonal field of view was used and connected to the Delft Remote Virtual Reality Exposure Therapy system (DRVRET; [35]) with virtual worlds that were visualized using a Vizard v3.0 software package.

#### iVET

Exposure exercises in iVET took place at the University of Amsterdam and its neighbourhood (e.g., supermarkets, subway stations, cafés, etc.), depending on the participant’s individual hierarchy of social situations. When relevant social situations could not be practiced at the University of Amsterdam or its neighbourhood, participants could carry out exposure exercises in their personal habitat substituting for a regular session. To keep these sessions comparable to regular sessions regarding structure and duration, participants contacted their therapist via phone before and after the exposure exercise.

### Procedure

The data used in the present study is part of a randomized controlled effectiveness trial on VRET and iVET for SAD that was carried out at the University of Amsterdam. A detailed description of the treatment and the analyses of treatment efficacy can be found in Kampmann et al. [25]. The results revealed that both VRET and iVET lead to significant reductions in SAD symptoms when compared to a waiting-list condition, with iVET being superior to VRET. The trial was approved by the Institutional Review Board of the University of Amsterdam and registered (NCT01746667; www.clinicaltrials.gov). After being contacted via the telephone, individuals filled in the SIAS online and were then invited for the in-person SCID-I intake if they scored above the SIAS cut-off. Individuals who did not meet any exclusion criteria as assessed during the intake were included, and informed consent was obtained. Participants were excluded in cases of a) psychotherapy for SAD in the past year; b) current use of tranquilizers or change in dosage of antidepressants in the past 6 weeks; c) a history of psychosis, current suicidal intentions, or current substance dependence; e) severe cognitive impairment; or f) insufficient command of the Dutch language. Eligible participants received a preassessment (LSAS-SR) and were then randomly assigned to either the VRET, iVET, or a wait-list condition using a computerized random number generator (http://www.randomization.com). Randomization and condition allocation was conducted by an individual not involved in the present study. Participants in the wait-list condition completed a second assessment after the waiting period and were then randomized to one of the treatment conditions. For the purpose of this study, we disregarded the wait-list condition such that data from participants who received treatment after the waiting period were pooled with the data from participants who were directly randomized to one of the treatment conditions. Before and after treatments sessions, participants filled in self-report questionnaires assessing potential predictors. For the purpose of this study, SCQ, SESS, and FAQ scores from only two time points (Time 1 and Time 2) were used to calculate change scores (see 2.2.3. for information on assessment points of each predictor variable). After treatment, all participant received a postassessment (LSAS-SR).

### Statistical analyses

The data were screened for outliers and normality. The residuals of the predictor variables were plotted for the inspection of nonlinearity and heteroscedasticity, but neither was constituted for any of the predictor variables. To deal with missing data, multiple imputation was applied, generating 20 separate datasets [36]. Next, for each of the predictors separately, a hierarchical multiple regression analysis was conducted including the preassessment of the outcome measure in step 1, the predictor in step 2, treatment condition and predictor × treatment condition in step 3, and LSAS as the outcome measure. Before calculating the product terms for the interaction effects, continuous predictor variables were mean centred [37]. Significant predictors (main effects) and moderators (interaction effects) of the individual predictor analyses (*p* < .05) were entered into a final multiple regression model assessing the effect of each predictor while controlling for the other predictors. As an indication of the unique contribution to the model, squared semipartial correlations (*sr*^2^) were calculated for each predictor. Beta weights and R^2^ values were separately calculated. We defined treatment drop-out as terminating therapy before at least 50% of the exposure sessions (i.e., at least six sessions) are reached. In literature, different definitions of treatment drop-out are used (Connell et al., [38]) and our decision was based on the expectation that some patients might not need to attend all ten sessions. This procedure has been also applied in other trials (A-Tjak et al., [39]; Masi et al., [40]). As reported in Kampmann et al. [25], treatment completers and dropouts did not significantly differ on demographic characteristics or outcome measures at preassessment. Furthermore, the completer sample did not significantly differed from the intent-to-treat sample in treatment efficacy. To increase power, data were pooled for participants receiving treatment directly and participants receiving treatment after the wait-list period since there was no significant difference between the two groups prior to starting treatment as reported in Table 3 in Kampmann et al. [25]. Furthermore, analyses were based on the aggregated sample of the two treatment groups because we aimed to investigate possible predictors of exposure therapy independent of treatment modality. However, to control for possible differences between the two groups, we included treatment condition as a moderator variable in the analyses.

## Results

See Table 1 for means and standard deviations of all predictor variables and Table 2 for regression equations. Individual predictor analyses revealed that a decrease in estimated social costs and self-focused attention in the first six sessions was associated with a better treatment outcome, as indicated by lower social fear and avoidance on the LSAS-SR. An increase in self-efficacy was also associated with lower social fear and avoidance. When including the main effect of treatment condition and the interaction between each predictor and treatment condition, the predictors did not remain significant in Step 3 of the individual predictor models. None of the predictors showed a significant interaction with the variable treatment condition. This indicates that the associations of social costs, self-focused attention, and self-efficacy with treatment outcome did not vary across treatment condition. The results of the final model, including all significant predictors from the initial individual predictor analyses, showed that only social costs remained a significant predictor of outcome on the LSAS-SR.

**Table 1:** Means and standard deviations for each predictor at the two time points

Measures	Group	Time 1		Time 2	
		M	SD	M	SD
FAQ	IVET	13.64	3.54	13.29	4.05
	VRET	13.10	4.61	12.7	4.43
SESS	IVET	39.36	6.56	50.08	12.63
	VRET	36.40	11.06	45.76	10.44
SCQ	IVET	112.57	19.77	96.83	30.18
	VRET	115.80	17.19	110.57	19.59

*Note. FAQ* Focus of Attention Questionnaire, *IVET* In Vivo Exposure Therapy, *SCQ* Social Costs Questionnaire, *SESS* Self-Efficacy Scale, *VRET* Virtual Reality Exposure Therapy

**Table 2:** Individual predictor and moderator analyses (a-c) and final model (d) with LSAS-SR as outcome measure

	B	SE B	β	*p*	sr^2^
a) Estimated social costs					
Step 1					
Constant	0.71	9.24		.938	
LSAS-SR pre	0.65	0.13	.56	**<.001**	.31
Step 2					
Constant	18.07	8.88		**.042**	
LSAS-SR pre	0.47	0.12	.40	**<.001**	.15
SCQ Δ	−0.41	0.08	−.52	**<.001**	.24
Step 3					
Constant	31.38	10.54		**.003**	
LSAS-SR pre	0.47	0.12	.41	**<.001**	.15
SCQ Δ	−0.41	0.34	−.52	.224	.02
Treatment	−9.01	4.59	−.42	.051	.04
SCQ Δ × treatment	0.03	0.19	.06	.894	.00
b) Self-focused attention					
Step 1					
Constant	0.71	9.24		.938	
LSAS-SR pre	0.65	0.13	.56	**<.001**	.31
Step 2					
Constant	2.30	8.81		.794	
LSAS-SR pre	0.64	0.13	.55	**<.001**	.30
FAQ Δ	−1.41	0.70	−.26	**.044**	.07
Step 3					
Constant	24.68	10.86		**.023**	
LSAS-SR pre	0.59	0.13	.51	**<.001**	.23
FAQ Δ	−0.33	1.97	−.06	.867	.00
Treatment	−13.39	4.46	−.62	**.003**	.10
FAQ Δ × treatment	−0.74	1.40	−.20	.598	.00
c) Self-efficacy					
Step 1					
Constant	0.71	9.24		.938	
LSAS-SR pre	0.65	0.13	.56	**<.001**	.31
Step 2					
Constant	13.43	9.70		.166	
LSAS-SR pre	0.57	0.13	.49	**<.001**	.23
SESS Δ	0.73	0.24	.34	**.003**	.11
Step 3					
Constant	30.13	12.01		.012	
LSAS-SR pre	0.52	0.13	.45	**<.001**	.16
SESS Δ	0.15	0.85	.07	.857	.00
Treatment	−12.73	4.43	−.59	**.004**	.09
SESS Δ × treatment	0.33	0.51	.26	.517	.01
d) Final model					
Step 1					
Constant	0.71	9.24		.938	
LSAS-SR pre	0.65	0.13	.56	**<.001**	.31
Step 2					
Constant	16.74	9.15		.068	
LSAS-SR pre	0.48	0.12	.41	**<.001**	.15
SCQ Δ	−0.34	0.12	−.43	**.001**	.09
FAQ Δ	−0.59	0.68	−.11	.438	.01
SESS Δ	−0.02	0.31	−.01	.825	.00

*Note.* a) *R*^*2*^ *=* .56; b) *R*^*2*^ *=* .39; c) *R*^*2*^ *=* .43; d) *R*^*2*^ *=* .57;

*FAQ* = Focus of Attention Questionnaire, *LSAS-SR* = Liebowitz Social Anxiety Scale-Self Report, *SCQ* = Social Costs Questionnaire, *SESS* = Self-Efficacy Scale, Δ = change scores

Significant *p*-values (*p* < .05) are marked in bold

## Discussion

The present study aimed at investigating a possible association between cognitions and treatment outcome in exposure therapy for SAD patients with heterogeneous social fears. For this purpose, we examined whether change during the first six sessions regarding self-focused attention, self-efficacy in social situations, and estimated social costs were associated with symptom change in individuals with SAD receiving exposure therapy. The results revealed that, when social costs, self-focused attention, and self-efficacy were separately examined, patients who showed a decrease in estimated social costs during the first six sessions reported a greater decrease of social anxiety symptoms after treatment and decreases in self-focused attention and self-efficacy during treatment were significantly associated with symptom improvement. When treatment condition (VRET and iVET) was part of the separate models, neither of the three predictors was significantly associated with treatment outcome. However, when social costs, self-focused attention, and self-efficacy were combined in one model, social costs were significantly associated with treatment outcome.

These findings are in line with earlier research that found decreases in social costs to be positively associated with treatment outcome in exposure therapy [19, 20]. However, while treatment in Smits et al. [20] focused on public speaking anxiety and treatment in Hofmann [19] was administered in a group format, our findings go further by showing that changes in expected social costs are associated with treatment outcome of individually administered exposure therapy in patients with SAD and heterogeneous social fears. Moreover, our results suggest that the effects of expected social costs as a predictor of treatment outcome might be consistent across different treatment modalities in exposure-based interventions. More specifically, in both in vivo and virtual reality exposure, declined estimated social costs seem to predict decrease of SAD symptoms.

Changes in self-focused attention and self-efficacy in social situations were significantly associated with treatment outcome when examined individually. Focussing less on oneself in social situations and gaining greater self-efficacy seem to foster improvement during exposure treatment. This was in accordance with research on cognitive behavioural therapy, showing that decreases in self-focused attention predict social anxiety reductions (see [9], for a review) and increases in self-efficacy are associated with treatment outcome of cognitive behavioural therapy [12, 13]. However, both variables were no longer significant predictors when they were part of the final model, indicating that neither one of them significantly contributed to the prediction of treatment outcome beyond social costs and treatment modality. Note, the inspection of the intercorrelations of the predictor variables suggests that social costs and self-efficacy might be partly overlapping concepts (*r* = −.66), but this applied less for social costs and self-focused attention (*r* = −.36).

The cognitive model of SAD [2] suggests that an individual who manages to focus less on himself/herself during exposure therapy, and therefore has less attention for anxiety symptoms, might report less SAD symptoms after treatment. Likewise, an individual who gains self-efficacy during treatment might feel less afraid of social situations after treatment and expect to better cope with social situations. However, if an individual learns during treatment to be less afraid of the negative consequences that his/her behaviour in social situations might have, he/she might report less fear and avoidance of social situations after treatment regardless of levels of self-focused attention and self-efficacy. In this situation, even if an individual cannot focus on the situation and does not think that he/she can handle the situation very well, he/she might be less afraid of the negative consequences that might follow. Learning that exposure to social situations is not followed by disastrous consequences and/or that individuals can cope with the (negative) consequences of their behaviour might help to decrease both anticipatory anxiety as well as anxiety during the actual social interaction. Yet, further research is needed to better understand the mechanism by which a reduction in expected social costs benefits a reduction of social anxiety symptoms. Moreover, one important question regarding clinical implications of these results is whether a stronger focus on expected social costs during exposure treatment can help to maximize treatment efficacy.

A limitation of the present study is the temporal overlap between the assessment of the predictor variables and the outcome measure given that we controlled for pre-treatment social anxiety in the analyses. While Hofmann [19] and Smits et al. [20] used mediation analyses, we investigated associations between the change in cognitions and treatment outcome rather than causal relationships. This was a first step in order to gather information on possible variables that could function as working mechanisms of exposure therapy. As suggested by Cole and Maxwell [41], for mediation analyses, multiple assessment points throughout treatment are necessary to investigate temporal precedence, which was not feasible within this study. Therefore, as a next step, future research should assess the session-to-session change in self-focused attention, self-efficacy, social costs and social anxiety levels within the framework of mediation analyses, to investigate changes in cognitions as a possible working mechanism of exposure therapy. Furthermore, not all predictor variables were assessed at the same time points. Whereas the self-efficacy and social costs measures referred to social situations in general and therefore could be assessed before the first exposure session, the self-focused attention measure referred to the social situations during the exposure exercises. Consequently, the first assessment of self-focused attention took place after the first exposure session, which was Session 3. However, the time between the assessment points was limited to one week because treatment was administered twice a week. Another limitation is that in addition to social cost bias, probability bias, the overestimation of the estimated probability that a negative social event occurs, was not assessed. A decrease of social cost bias might have resulted from a reduction of probability bias as suggested by Smits et al. [20]. Given the equivocal results on the predominance of social cost and probability bias in the literature, future research is needed that simultaneously examines social cost and probability bias as predictors of treatment outcome after exposure therapy for SAD. Finally, we can only tentatively conclude that the effects of the investigated predictors on treatment outcome do not differ between in vivo and virtual reality exposure therapy, since we cannot rule out that insufficient power is responsible for the lack of differential effects.

## Conclusions

The current study suggests that changes in estimated social costs are associated with improvement of social anxiety symptoms after exposure therapy. Thus, individuals who report greater changes of estimated social costs during treatment may benefit more from treatment, as was the case for both in vivo and virtual reality exposure therapy. Changes in self-focused attention and self-efficacy during treatment were not significantly associated with symptom improvement anymore when examined in combination with social costs. This suggests that self-focused attention and self-efficacy did not predict symptom improvement beyond social costs. Future research needs to further investigate estimated social costs as a predictor in relation to other cognitions in SAD patients with heterogeneous social fears and whether maximizing decreases in estimated social costs can increase treatment efficacy.

## Abbreviations

FAQ: Focus of Attention Questionnaire
iVET: in vivo exposure therapy
LSAS-SR: Liebowitz Social Anxiety Scale-Self Report
SAD: social anxiety disorder
SCID-I: Structured Clinical Interview
SCQ: Social Costs Questionnaire
SESS: Self-Efficacy for Social Situations Scale
SIAS: Liebowitz Social Anxiety Scale-Self Report
VRET: virtual reality exposure therapy

## References

[1] American Psychiatric Association (2013). Diagnostic and statistical manual of mental disorders.

[2] Clark DM, Wells A, Heimberg RG, Liebowitz MR, Hope DA, FRS (1995). A cognitive model of social phobia. Soc. phobia Diagnosis, assessment, Treat.

[3] Acarturk C, Cuijpers P, van Straten A, de Graaf R (2009). Psychological treatment of social anxiety disorder: A meta-analysis. Psychol Med.

[4] Feske U, Chambless DL (1995). Cognitive behavioral versus exposure only treatment for social phobia: A meta-analysis. Behav Ther.

[5] Powers MB, Sigmarsson SR, Emmelkamp PMG (2008). A meta–analytic review of psychological treatments for social anxiety disorder. Int J Cogn Ther.

[6] Rapee RM, Heimberg RG (1997). A cognitive-behavioral model of anxiety in social phobia. Behav Res Ther [Internet].

[7] Chen J, Rapee RM, Abbott MJ. Mediators of the relationship between social anxiety and post-event rumination. J. Anxiety Disord. [internet] Elsevier Ltd; 2013;27:1–8. Available from: 10.1016/j.janxdis.2012.10.008. 10.1016/j.janxdis.2012.10.008 23247198

[8] Wong QJJ, Gregory B, McLellan LF, Kangas M, Abbott MJ, Carpenter L, McEvoy PM, Peters L, Rapee RM (2017). Anticipatory processing, maladaptive attentional focus, and postevent processing for interactional and performance situations: treatment response and relationships with symptom change for individuals with social anxiety disorder. Behav Ther.

[9] Norton AR, Abbott MJ. Self-Focused Cognition in Social Anxiety: A Review of the Theoretical and Empirical Literature. Behav. Chang. [Internet]. 2016;33:44–64. Available from: http://www.journals.cambridge.org/abstract_S0813483916000024.

[10] Hofmann SG (2000). Self-focused attention before and after treatment of social phobia. Behav Res Ther.

[11] Thomasson P, Psouni E (2010). Social anxiety and related social impairment are linked to self-efficacy and dysfunctional coping. Scand J Psychol.

[12] Gaudiano BA, Herbert JD (2007). Self-efficacy for social situations in adolescents with generalized social anxiety disorder. Behav Cogn Psychother.

[13] Gaudiano BA, Herbert JD (2003). Preliminary psychometric evaluation of a new self-efficacy scale and its relationship to treatment outcome in social anxiety disorder. Cogn Ther Res.

[14] Delsignore A, Carraro G, Mathier F, Znoj H, Schnyder U. Perceived responsibility for change as an outcome predictor in cognitive-behavioural group therapy. Br. J Clin Psychol [Internet]. 2008;47:281–293. Available from: http://www.ncbi.nlm.nih.gov/pubmed/18248693 10.1348/014466508X279486 18248693

[15] Foa EB, Kozak MJ, Tuma AH, Maser JD (1985). Treatment of anxiety disorders: implications for psychopathology. Anxiety anxiety Disord. Erlbaum, Hillsdale, NJ.

[16] McManus F, Clark DM, Hackmann A (2000). Specificity of cognitive biases in social phobia and their role in recovery. Behav Cogn Psychother.

[17] Calamaras MR, Tully EC, Tone EB, Price M, Anderson PL. Evaluating changes in judgmental biases as mechanisms of cognitive-behavioral therapy for social anxiety disorder. Behav. Res. Ther. [internet]. Elsevier Ltd; 2015;71:139–49. Available from: http://linkinghub.elsevier.com/retrieve/pii/S0005796715001059. 10.1016/j.brat.2015.06.006 PMC4509570 26140823

[18] Foa EB, Franklin ME, Perry KJ, Herbert JD. Cognitive biases in generalized social phobia. J Abnorm Psychol [Internet]. 1996;105:433–439. Available from: https://psycnet.apa.org/record/1996-00463-013. 8772013

[19] Hofmann SG (2004). Cognitive mediation of treatment change in social phobia. J Consult Clin Psychol.

[20] Smits JAJ, Rosenfield D, McDonald R, Telch MJ. Cognitive mechanisms of social anxiety reduction: an examination of specificity and temporality. J Consult Clin Psychol [Internet]. 2006;74:1203–12. Available from: http://www.ncbi.nlm.nih.gov/pubmed/17154749 10.1037/0022-006X.74.6.1203 17154749

[21] Rapee RM, Gaston JE, Abbott MJ (2009). Testing the efficacy of theoretically derived improvements in the treatment of social phobia. J Consult Clin Psychol.

[22] Gregory B, Peters L, Abbott MJ (2015). Relationships between probability estimates, cost estimates, and social anxiety during cbt for social anxiety disorder. Cogn Ther Res.

[23] Hedman E, Mörtberg E, Hesser H, Clark DM, Lekander M, Andersson E, Ljótsson B (2013). Mediators in psychological treatment of social anxiety disorder: individual cognitive therapy compared to cognitive behavioral group therapy. Behav Res Ther.

[24] American Psychiatric Association (2000). Diagnostic and statistical manual of mental disorders.

[25] Kampmann IL, Emmelkamp PMG, Hartanto D, Brinkman W-P, Zijlstra BJH, Morina N. Exposure to virtual social interactions in the treatment of social anxiety disorder: A randomized controlled trial. Behav. Res. Ther. [Internet]. 2016;77:147–56. Available from: http://www.sciencedirect.com/science/article/pii/S0005796715300802. 10.1016/j.brat.2015.12.016 26752328

[26] Mattick R, Clarke C (1998). Development and validation of measures of social phobia scrutiny fear and social interaction anxiety. Behav Res Ther.

[27] First MB, Spitzer RL, Gibbon M, Williams JBW. Structured Clinical Interview for Axis I DSM–IV Disorders - Research ed. New York, NY: Biometrics Research Department; 1994.

[28] Liebowitz MR (1987). Social phobia. Mod Probl Pharmacopsychiatry.

[29] Baker SL, Heinrichs N, Kim HJ, Hofmann SG (2002). The Liebowitz social anxiety scale as a self-report instrument: A preliminary psychometric analysis. Behav Res Ther.

[30] Woody SR (1996). Effects of focus of attention on anxiety levels and social performance of individuals with social phobia. J Abnorm Psychol.

[31] Woody SR, Chambless DL, Glass CR (1997). Self-focused attention in the treatment of social phobia. Behav Res Ther.

[32] Hofmann SG, Otto MW (2008). Cognitive-behavior therapy of social anxiety disorder: evidence-based and disorder specific treatment techniques.

[33] Scholing A, Emmelkamp PMG (1993). Exposure with and without cognitive therapy for generalized social phobia: effects of individual and group treatment. Behav Res Ther.

[34] Brinkman W-P, Hartanto D, Kang N, de Vliegher D, Kampmann IL, Morina N, et al. A virtual reality dialogue system for the treatment of social phobia. Proc. ACM CHI ‘12 Ext. Abstr. Hum. Factors Comput. Syst. USA [Internet]. 2012;1099–1102. Available from: http://dl.acm.org/citation.cfm?id=2212776.2212395.

[35] ter Heijden N, Brinkman W-P. Design and evaluation of a virtual reality exposure therapy system with automatic free speech interaction. J CyberTherapy Rehabil [Internet] 2011;4:41–56.

[36] Sterne J A C, White IR, Carlin JB, Spratt M, Royston P, Kenward MG, et al. Multiple imputation for missing data in epidemiological and clinical research: potential and pitfalls. Br. Med. J. [Internet]. 2009;338:b2393. Available from: http://www.bmj.com/cgi/doi/10.1136/bmj.b2393. 10.1136/bmj.b2393 PMC2714692 19564179

[37] Tabachnick BG, statistics FLSU m. Using Multivar. Stat 5th ed. 2007:980.

[38] Connell J, Grant S, Mullin T (2006). (2006). Client initiated termination of therapy at NHS primary care counseling services. Counseling and Psychotherapy Research.

[39] A-Tjak JG, Morina N, Topper M, Emmelkamp PM (2018). A randomized controlled trial in routine clinical practice comparing acceptance and commitment therapy with cognitive behavioral therapy for the treatment of major depressive disorder. Psychotherapy and Psychosomatics.

[40] Masi MV, Miller RB, Olson MM (2003). Differences in dropout rates among individual, couple, and family therapy clients. Contemporary Family Therapy.

[41] Cole DA, Maxwell SE (2003). Testing mediational models wih longiturdinal data: questions and tips in the use of structurra equation modeling. J Abnorm Psychol.

